# A Rare Case of Valproic Acid Toxicity Requiring Hemodialysis

**DOI:** 10.7759/cureus.41020

**Published:** 2023-06-27

**Authors:** Nyier W Doar, Samaj Adhikari, Binit Aryal, Sushma Edara, Marie Schmidt

**Affiliations:** 1 Internal Medicine, Interfaith Medical Center, Brooklyn, USA; 2 Pulmonary and Critical Care Medicine, Interfaith Medical Center, Brooklyn, USA

**Keywords:** acute poisoning, extracorporeal treatments, valproate overdose, cardiac arrythmia, respiratory depression, hd ( hemodialysis ), valproic acid toxicity

## Abstract

Valproic acid poisoning can have mild to fatal consequences depending on its body concentration. There are rare case reports and barely any known controlled studies on the use of hemodialysis as a last treatment resort. We report a rare valproic acid poisoning case at One Brooklyn Health/Interfaith campus, New York City, warranting intubation and hemodialysis.

The patient is a 47-year-old male with a past medical history of seizure disorder, polysubstance use disorder, schizophrenia, and gastroesophageal reflux disease (GERD) who was brought to the medical emergency department (ED) for intentional valproic acid overdose with 60 tablets of his prescribed home Depakote DR 500 mg (~30 g). The patient’s other outpatient medications included valproic acid, trazodone, acetaminophen, famotidine, fluoxetine, folic acid, hydrocortisone-aloe, multivitamin, nicotine polacrilex, and thiamine. The patient’s initial blood tests showed high valproic acid, ammonia, ethanol, and lactate. About six hours after ED admission, the patient became somnolent, desaturated to 74% on a non-rebreather oxygen mask, warranting intubation and hemodialysis after noticing persistently high serum concentrations of valproic acid.

The relatively low molecular weight (144 Daltons) and low volume of distribution of valproic acid suggest a potential benefit from hemodialysis, especially at a serum concentration of >850 mg/L or in the event of a shock. In this patient, mentation and stability status were improved after hemodialysis. Hemodialysis appears to be the last treatment resort for severe valproic acid poisoning.

## Introduction

This article was previously presented as a conference poster at 2022 One Brooklyn Health Eighth Annual Research Day on November 16, 2022.

Valproic acid (2-propylpentanoic acid; VPA) is a branched-chain carboxylic acid that was first introduced as an anti-epileptic drug but has also been used to manage bipolar disorder and migraine headaches. Overdosing of VPA can have several consequences, ranging from mild central neurological depression to death. The most common acute manifestation of VPA poisoning is lethargy followed by uneventful recovery [1]. Other common manifestations include respiratory depression, arrhythmias, hypotension, hyperammonemia, anion gap metabolic acidosis, thrombocytopenia, hepatitis, diarrhea, and cerebral edema [2].

There are several management modalities for various VPA toxicities as described by Sztajnkrycer [3]. While most of these management strategies have been frequently employed to treat VPA toxicities, toxicities that warrant hemodialysis are rare and there are no available control studies yet for it [4]. We report a rare case of a patient at Interfaith Medical Center in Brooklyn, New York, who presented with acute VPA poisoning warranting hemodialysis and intubation. We further discussed some of the recent guidelines developed by the extracorporeal treatments in poisoning (EXTRIP) on circumstances that may meet the criteria for hemodialysis and shed light on why hemodialysis may be the only last resort of intervention in such cases.

## Case presentation

A 47-year-old male patient with past medical histories of seizure disorder, polysubstance use disorder, schizophrenia, and gastroesophageal reflux disease (GERD) was brought to the medical emergency department (ED) for intentional VPA overdose. The patient stated he took 60 tablets of his prescribed Depakote DR 500 mg (~30 g) about 1.5 hours before arrival in the ED. The patient said he took the pills because he was “feeling suicidal.” The patient also mentioned taking some alcohol the same night he took the pills but could not state the amount. On review of systems, the patient denied shortness of breath, chest pain, fever, palpitations, and vision changes, but endorsed depression.

The patient’s outpatient medications included VPA, trazodone, acetaminophen, famotidine, fluoxetine, folic acid, hydrocortisone-Aloe, multivitamin, nicotine Polacrilex gum, and thiamine. He is allergic (gets hives) to celecoxib and esomeprazole. The patient is a current everyday smoker and alcohol drinker and uses cocaine. He used to live at a shelter but is currently homeless.

Patient’s triage vital signs showed saturation of 98% while breathing ambient air, oral temperature of 36.8 degree Celsius, heart rate of 76/minute, blood pressure of 133/72, and respiratory rate of 16/minute. The patient appeared obese, otherwise in no respiratory distress. All other physical exams were unremarkable. 

**Figure 1:**
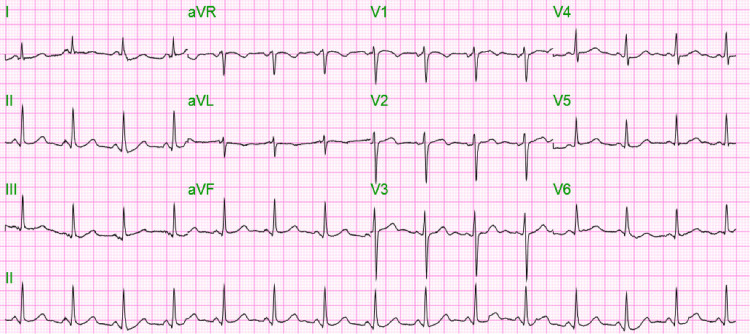
Initial EKG at admission. Arrhythmias ruled out.

**Figure 2:**
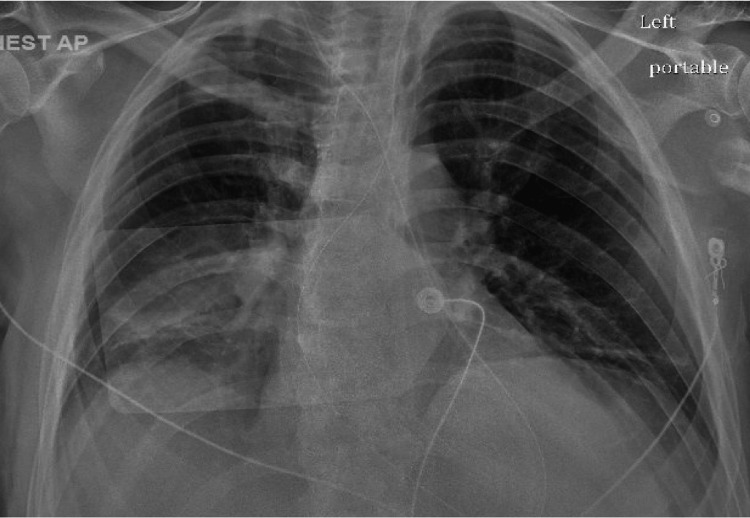
Initial chest x-ray, neg for any acute pathology.

**Figure 3:**
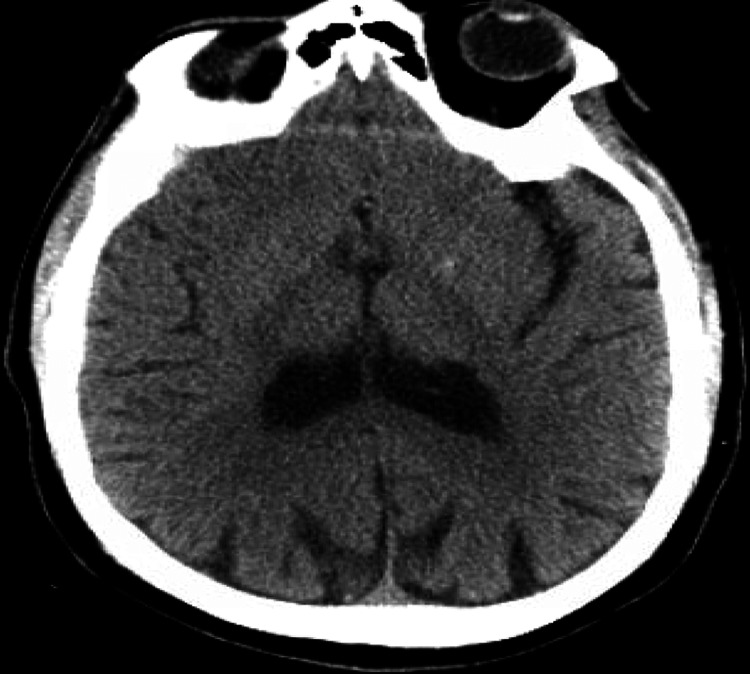
Initial CT head without contrast: no cerebral edema.

The patient’s initial significant abnormal laboratory findings were as summarized in Table 1, showing elevated serum VPA, ammonia, ethanol, and lactate. His initial electrocardiogram, chest x-ray, and head computed tomography (CT) scan did not reveal any acute pathologies as displayed in Figures 1-3.

**Table 1:** Patient's initial laboratory findings on admission.

Investigation	Value	Reference range
Valproic acid	>300	50-100 µg/mL
Ammonia	110.9	18.0-72.0 µmol/L
Ethanol	257.19	0.00-14.00 mg/dL
Urine	Benzos	neg
Lactate	4.55	0.05-1.90 mmol/L

Given these initial evaluation results, the patient was kept in the ED for observation. About three hours after ED admission, the patient became increasingly somnolent, groggily opened his eyes to touch. Patient started desaturating on room air and was placed on a non-rebreather (NRB) oxygen mask. The Poison Control unit was contacted, and the patient was started on levocarnitine injection. About six hours later, the patient desaturated to 74% on NRB; his lactate increased to 6.6 and ammonia increased to 279 while his VPA levels remained constantly above 300 µg/mL. Patient had to be intubated and admitted to the intensive care unit (ICU). Patient then got emergency dialysis which brought back the serum VPA levels to subtherapeutic levels and resolved the lactic acidosis and hyperammonemia. Patient was later weaned and discharged to the psychiatry unit for further management.

## Discussion

VPA is extensively used as an anticonvulsant, acute mood stabilizer and to treat migraines. VPA works by increasing gamma-aminobutyric acid (GABA), blocking voltage-gated sodium channels, thus causing CNS depression, arrhythmia and disrupting the urea cycle, causing hyperammonemia [5]. VPA absorption depends on its preparation forms. In our case, the patient took enteric coated delayed release VPA. VPA has high protein binding of 90%-95% with a half-life of 10-20 hours, while its acceptable therapeutic range of total VPA is 50-100 µg/m [6]. At higher concentrations, the plasma binding capacity becomes saturated, resulting in higher unbound VPA, accounting for its toxicities.

**Figure 4:**
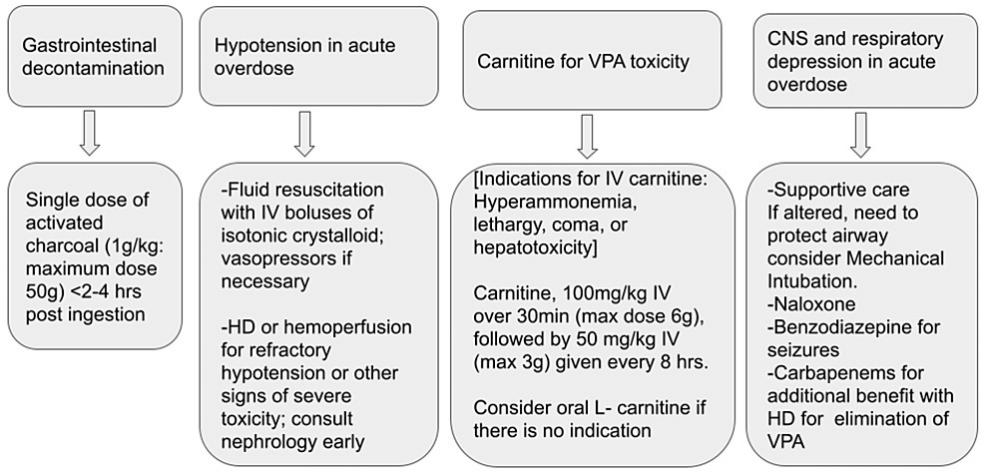
Common treatment modalities for VPA toxicities other than hemodialysis. VPA - valproic acid

Management of VPA poisoning is largely supportive, especially with activated charcoal which decreases its absorption [6]. Most common medical management involves early IV Levocarnitine which plays an important role in hyperammonemia encephalopathy and hepatotoxicity by eliminating ammonia as urea and glutamine [7]. Other common medical management strategies are summarized in Figure 4.

There are rare case reports and no known controlled studies on the use of hemodialysis as a last treatment resort for VPA poisoning, yet there are recent reports that toxic concentrations of VPA can be excreted effectively with Hemodialysis [7]. The relatively low molecular weight (144 Daltons) and low volume of distribution of VPA suggest a potential benefit from hemodialysis compared to the usual treatment modalities summarized in Figure 4. Per recent guidelines developed by the EXTRIP, circumstances that may meet the criteria for hemodialysis include serum VPA concentration of >850 mg/L, cerebral edema, or in the event of a shock [4]. With serum levels more than 850 μg/mL, hypotension, cardiac arrest, and respiratory depression requiring intubation have been reported [4]. While our hospital laboratory facility does not give an exact concentration of serum VPA for any value above 300 mg/L, the fact that repeated measurements before dialysis were persistently above 300 mg/L most likely indicate concentrations above 850 mg/L. Right after our patient got emergency dialysis which brought back the serum VPA levels to subtherapeutic levels and resolved the lactic acidosis and hyperammonemia; the patient's mentation and hemodynamic status were improved and was later weaned off the ventilator and discharged.

The main limitation in this case report is lack of sufficient randomized control trials to confirm the efficacy of hemodialysis in reversing the VPA toxicities. As per Ghannoum et al. [4], there are barely any available control studies yet for VPA toxicities that warrant hemodialysis.

## Conclusions

In short, the most common acute manifestation of VPA poisoning is lethargy followed by uneventful recovery. VPA causes CNS depression by increasing the GABAergic blockage of voltage-gated sodium channels. Hemodialysis appears to be the last treatment resort for severe VPA poisoning. Circumstances that warrant VPA hemodialysis as outlined by EXTRIP include VPA serum concentration of >850 mg/L, cerebral edema, and shock event.
